# “Beauty Is How You Feel Inside”: Aesthetic Judgments Are Related to Emotional Responses to Contemporary Music

**DOI:** 10.3389/fpsyg.2020.510029

**Published:** 2020-11-13

**Authors:** Hauke Egermann, Federico Reuben

**Affiliations:** York Music Psychology Group, Music Science and Technology Research Cluster, Department of Music, University of York, York, United Kingdom

**Keywords:** music, emotion, aesthetic judgment, psychophysiology, contemporary music, concert

## Abstract

While it has extensively been argued that aesthetic categories such as beauty have a direct relationship to emotion, there has only been limited psychological research on the relationship between aesthetic judgments and emotional responses to art. Music is recognized to be an art form that elicits strong emotional responses in listeners and it is therefore pertinent to study empirically how aesthetic judgments relate to emotional responses to music listening. The aim of the presented study is to test for the impact of aesthetic judgment on various psychophysiological response measures of emotion that were assessed in parallel in two contemporary music concerts, each with a different audience and program. In order to induce different levels of aesthetic judgments in participants, we assigned them randomly to one of two groups in a between-subjects design in both concerts: One group attended a talk on the music presented, illustrating its aesthetic value, while the other group attended an unrelated talk on a non-musical topic. During the concerts, we assessed, from 41 participants in Concert 1 (10 males; mean age 23 years) and 53 in Concert 2 (14 males; mean age 24 years), different emotional response components: (a) retrospective rating of emotion; (b) activation of the peripheral nervous system (skin conductance and heart rate); (c) the activity of two facial muscles associated with emotional valence (only Concert 1). Participants listened to live performances of a selection of contemporary music pieces. After each piece, participants rated the music according to a list of commonly discussed aesthetic judgment criteria, all thought to contribute to the perceived aesthetic value of art. While preconcert talks did not significantly impact value judgment ratings, through factor analyses it was found that aesthetic judgments could be grouped into several underlying dimensions representing analytical, semantic, traditional aesthetic, and typicality values. All dimensions where then subsequently shown to be related to subjective and physiological responses to music. The findings reported in this study contribute to understanding the relationship between aesthetic judgment processes and emotional responses to music. The results give further evidence that cognitive-affective interactions have a significant role in processing music stimuli.

## Introduction

“Beauty is how you feel inside, and it reflects in your eyes.” is the first sentence of a well-known quote by actress Sofia Loren (Green, 1982, p. 340) that has been assimilated in popular culture for its self-evident association with the idea of *inner beauty* and that it is our *inner traits*, and not our *physical attributes*, that makes us, as a person, *beautiful*. However, if this sentence is re-contextualized, and taken at face value, it can have a different and perhaps more complex and deeper philosophical meaning, as it can suggest that there is an inherent link between the aesthetic (beauty) and the emotional (inside feelings). While it has extensively been argued that aesthetic categories such as beauty have a direct relationship to emotion (Juslin, 2013; Schindler et al., 2017), there has only been limited psychological research on the relationship between aesthetic judgments and emotional responses to art. Music is recognized to be an art form that gives strong emotional responses to listeners (Koelsch, 2010) and it is therefore pertinent to study empirically how aesthetic judgments are associated with emotional responses to music listening. This article reports the results of two concert experiments that will contribute to the understanding of how the aesthetic value listeners place in music is related to their emotional response to it. The study focuses on audiences listening to contemporary music that some listeners might describe as ‘difficult’ or ‘challenging,’ as some of this music has features that, according to several theories of emotional processing (Scherer and Coutinho, 2013), can be associated with negative emotion. At the same time, this music might be enjoyable to some other listeners, pointing to the presupposition that in such cases, aesthetic judgments might influence their emotional responses to the music. Challenging contemporary music could therefore be particularly well-suited as a stimulus for studying the link between aesthetic judgments and emotion.

### Music and Emotion

*Emotion*, as defined in this study, can be understood through Scherer’s (2005) *component process model*, which states that an emotional episode consists of coordinated changes in three major reaction components: (a) physiological arousal, (b) motor expression and (c) subjective feelings, all driven by cognitive appraisal triggered by an emotional stimulus. Measuring emotional reactions to music should, therefore, capture all three different response components at the same time. Recent studies give further evidence that changes in these three reaction components can be induced by music. For example, Lundqvist et al. (2008) demonstrate that music can induce feelings of happiness or sadness with associated activations of the autonomic nervous system (measured through skin conductance), and activations of expressive facial muscles. Grewe et al. (2009) show that strong emotional responses to music like the *chill* response (experience of shivers or goose bumps) are accompanied by increases in felt emotional intensity, skin conductance, and heart rate (HR). Furthermore, a study by Salimpoor et al. (2011), gives evidence that strong music-induced emotions are manifested neurochemically, by dopamine release in the reward system in the human brain, in a similar manner to other pleasurable stimulations like food intake, sex, or drugs.

Several psychological theoretical frameworks exist that aim to explain emotional responses to music. For example, Juslin et al. (2010) summarize different theories on emotion-induction and apply them to music (see also Scherer and Zentner, 2001). According to this model, the following psychological mechanisms are involved in music listening: *cognitive appraisal, evaluative conditioning*, *episodic memory, musical expectation, emotional contagion/empathy, visual imagery, brain stem reflexes* and *rhythmic entrainment*. A multitude of experimental research on these specific functions of individual emotion-induction mechanisms has been conducted (for a review, see Egermann and Kreutz, 2018). The findings of these studies show that when musicians express emotion through music, they make use of acoustic features similar to those used in other modalities of behavior such as human vocal expression (Juslin and Laukka, 2003) or sounds produced during walking (Giordano et al., 2014). For example, the expression of negative emotions such as fear and anger, has been shown to be associated with high tempo, absolute sound level, sound level and pitch variability, and high-frequency energy. In Egermann and McAdams (2013), it was shown that music that is rated to be expressive of high or low arousal and positive or negative valence leads to corresponding induced emotions through emotional contagion and when listeners indicate that they empathize with the music they hear. Steinbeis et al. (2006), demonstrated that harmonic expectancy violations lead to corresponding increases in continuous intensity and tension ratings, as well as skin conductance (see also Egermann et al., 2013). While most of these studies were conducted in laboratory settings where participants listened to pre-recorded music alone, only a small number of studies measured the emotional responses of an audience listening to music performed in an ecologically valid live setting (McAdams et al., 2004; Stevens et al., 2009; Thompson, 2006; Egermann et al., 2013).

### Aesthetic Judgment and Music

Various theories within philosophical aesthetics provide a different perspective for understanding listeners’ responses to music. These theories often describe the value of music and art through different *aesthetic judgment criteria* such as the representation of nature; having features such as beauty, complexity or sublimity; being expressive, original, tasteful, or prototypical; showing artistic skill; conveying messages; or being defined as valuable by institutions (for a review, see Juslin, 2013). Furthermore, the field of *new experimental aesthetics* (Berlyne, 1971) empirically investigates aesthetic responses to various forms of art. Leder et al. (2004), for example, propose a model of aesthetic experience that suggests several sequential processes such as stimulus classification as art, perceptual analyses, memory integration and cognitive mastering that inform aesthetic judgments of art.

Traditionally, research in music psychology has focused on understanding listeners’ emotional responses, while research in experimental aesthetics has focused on aesthetic judgments of value and aesthetic experiences in the arts (Leder et al., 2004). Recently the two research traditions have been integrated into a common model. In 2013, Juslin proposed a further emotion-induction mechanism that he termed *aesthetic judgment*. He proposed that when music is experienced within an artistic frame, like a concert, aesthetic judgments are triggered based on criteria like beauty, expression, originality, skilfulness, or typicality. While some judgment criteria can be related to a traditional Kantian understanding of aesthetics as not specific to art (e.g., beauty, the sublime), others can be considered according to a more contemporary understanding of artistic value (for example, artistic innovation and originality; conceptual depth; and artistic value (re)defined by institutions, artists and the art market). This differentiation, we would like to suggest, points to the idea that there might be two types of judgment values associated with the reception art that can also be linked with different mental processes. On the one hand, there might be a link between aesthetic value and affective experience, and on the other hand, between artistic value and cognitive engagement with art. From Juslin’s (2013) theory, it can be deduced that judging a piece of music as having high aesthetic and/or artistic value will induce positive emotional responses. However, the exact underlying affective and cognitive mechanisms involved in aesthetic judgments still remain unclear.

For the purpose of this study, *aesthetic judgments* can be defined as value assessments based on various aesthetic judgment criteria. These judgment criteria may be based on socially constructed cognitive appraisals (e.g., ‘This piece of music has high value to me because it was skilfully composed and is meaningful to me’) or affective experiences (‘This piece of music has high value because it is very expressive and touches me’).

Aesthetic judgments are closely related to concepts such as liking or preference, but they are not equal to them. If a piece of music is of high value to a listener, they are more likely to prefer or like it. However, music preferences are not only influenced by aesthetic judgments, they can also be influenced by other factors such as familiarity and social identity (Lamont and Greasley, 2009). Aesthetic judgments are conceived as conscious decision-making processes and studying them could contribute to understanding the underlying cognitive-affective interactions shaping musical experience. Therefore, aesthetic judgment could be similar to general cognitive appraisal of goal congruency (Scherer, 2005) and emotional reappraisal, which has been suggested to influence emotion regulation in general (Gross, 2002).

### Contemporary Music

Philosopher Robinson (2005) has discussed the importance of music that not just simply provokes an emotional response in listeners, but that produces complex and ambiguous emotions that actively encourage them to reflect about, and learn from, their listening experience. Contemporary music often produces this kind of emotional response, and at the same time, has a reputation of being ‘challenging’ or ‘difficult’ to new audiences in part for the complex emotions it evokes and the novelty of its ideas, techniques and materials. However, listeners who actively engage with this music report that it is an enjoyable, stimulating and educational experience that enriches them emotionally and intellectually (Gross and Pitts, 2016). Furthermore, understanding the mechanisms behind the creation of contemporary music has been shown to be associated with an increase in positivity of audience experiences (Emerson and Egermann, 2018).

While most experimental research on emotional responses to contemporary music has focused on stimulus characteristics (e.g., McAdams et al., 2004; Bailes and Dean, 2007), this study focuses on the relationship between aesthetic judgments and psychophysiological emotional responses in listeners. There are several mechanisms of emotional processing of music, including emotional contagion, musical expectation, or brain stem reflexes (Juslin et al., 2010) that might explain why contemporary music that is complex, dissonant, or loud can induce negative emotional responses. However, at the same time, for some listeners this music can be enjoyable, and we hypothesize that this might be because aesthetic value judgments may positively influence their emotional responses to it. This makes contemporary music particularly suitable for studying the interaction of cognitive and affective systems involved in music listening. In other words, challenging contemporary music may cause the affective system to respond with negative emotions due to difficult stimulus characteristics, and, at the same time, the cognitive system to generate positive emotions due to the artistic value identified in the music. Studying aesthetic value judgments and emotional responses to contemporary music, therefore, may allow for a better understanding of the interaction of cognitive and affective systems involved in music listening as the different mechanisms might create divergent responses.

### Aims

The aim of this study is to examine the impact of aesthetic judgment on various psychophysiological response measures of emotion. Aesthetic judgments and emotional responses were assessed in parallel and tested in two live concerts with two different audiences listening to contemporary music. Conducting this research in ecologically valid settings allowed the presentation of the music to occur within an artistic frame that was hypothesized to trigger aesthetic judgment processes.

**FIGURE 1:**
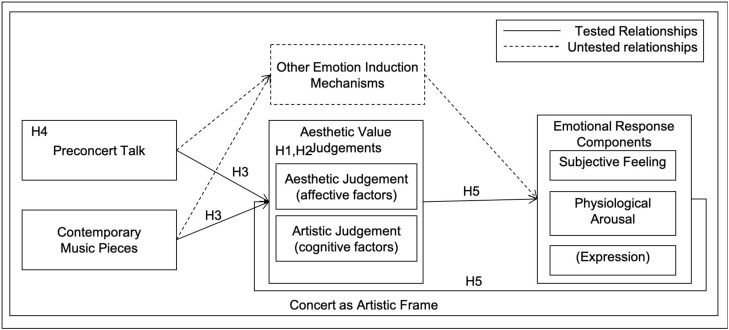
Theoretical model tested in this study with individual hypotheses.

Previous research suggests that judgments of musical characteristics can be influenced through information presented to participants prior to music listening (Fischinger et al., 2018). In order to evoke different levels of aesthetic judgments in participants (and test for a causal effect of aesthetic judgment on emotion), we assigned them randomly to one of two groups in a between-subjects design. Each group attended a preconcert talk on a different subject: one on the music presented, highlighting its aesthetic value (experimental group); and the other on an unrelated non-musical topic (control group). This design was repeated in two separate concerts with different participants. Based on the theoretical and empirical work previously reviewed, we postulated the following hypotheses (see also Figure 1):

• H_1_: Aesthetic and artistic judgments based on individual criteria items can be grouped into different underlying affective and cognitive aesthetic judgment factors (AJFs)
• H_2_: AJFs are associated with manifest aesthetic and artistic value ratings
• H_3_: Different pieces of music and a preconcert talk evoke different levels of AJFs
• H_4_: AJFs mediate between the effect of a preconcert talk on emotional responses scores
• H_5_: Cognitive and affective AJFs are associated with emotional response components

## Methods

### Participants

For Concert 1, we recruited 41 participants who were all students at the University of York. They were screened with the help of an online questionnaire before taking part to ensure that they had some familiarity with, and preference for, classical music; would show willingness to be filmed; and were willing to shave (only males, due to facial electrode placement). Their mean age was 23 years, range 18–42 years (10 males). 18 identified themselves as music students and 23 as non-music students. For Concert 2, we subsequently recruited 53 participants (14 males; mean age 24 years) who were all non-music students nor professional musicians. All were also students at the University of York and were selected as well for having some preference for classical music, but not specifically for contemporary or experimental music.

### Stimuli

All the pieces of music presented as stimuli were performed live, in front of the audience or, if they contained electroacoustic materials, reproduced via two Genelec 1037C speakers (see Table 1). We chose the stimuli for Concert 1, based on the following criteria: (a) they presumably contained features that are typically difficult to appreciate (e.g., high complexity, low semantic clarity), (b) they had contrasting music styles and characteristics between each other, and (c) they could be performed by students or members of staff in Department of Music. For Concert 2, one of the authors who is an expert in contemporary music selected seven contemporary piano music pieces that each were hypothetically associated with one of the seven different underlying aesthetic emotion factors included in the Aesthetic Emotions Scale (AESTHEMOS) (Schindler et al., 2017). This was done in order to assure that the music presented in the concert would cover a wide range of emotional states. Furthermore, the music had to be within the repertoire of the professional pianist who performed the pieces in Concert 2.

**TABLE 1:** Music pieces performed in concerts.

**Order**	**Composer**	**Title**	**Performer**	**Instrumentation**
**Concert 1**				
1	Neil Luck	Things	James Mcilwrath	Percussion on Table
2	Karlheinz Stockhausen	Klavierstück IX	Anson Ng	Piano
3	Pauline Oliveros	Bye Bye Butterfly	n/a	Electroacoustic composition for fixed tape
4	Improvisation	n/a	Mainwaring/Reuben Duo	Saxophone and live coding/laptop
**Concert 2**				
1	György Ligeti	Musica Ricercata I, III, IV		
2	Karlheinz Stockhausen	Klavierstücke VII		
3	György Ligeti	Arc-en-ciel (Études Book 1)		
4	Helmut Lachenmann	Guero	Kate Ledger	Piano
5	George Crumb	A Little Suite for Christmas II, III, IV, XI		
6	Steve Martland	Snapshot		
7	Michael Finnissy	Our Love Is Here To Stay		

### Measurements

As audience response measurements, we assessed in both concerts three different emotional response components (subjective feeling, physiological arousal, and expressive behavior), as well as aesthetic judgments.

#### Subjective Feelings and Aesthetic Judgments

For Concert 1, we used the 25-item version of the Geneva Emotion Music Scales (Zentner et al., 2008) and a self-developed aesthetic judgment questionnaire, including various items used in previous research that represent different categories of aesthetic judgment criteria (Table 2). We identified several of those categories from studies by Juslin and colleagues (Juslin, 2013; Juslin and Isaksson, 2014; Juslin and Västfjäll, 2008). We decided to not use the following categories from Juslin and Isaksson (2014): *Use as Art, Representation, Artistic intention, Wittiness* because they received rather low importance ratings with regards to their relevance influencing participant’s music choices and were considered as less relevant in the context of the contemporary music repertoire presented. We took Items 1, 2, 3, 8 as used in Juslin and Västfjäll (2008), and added Items 4, 5, 6, 7, 9, reflecting the same criteria categories from studies by Juslin and colleagues. Furthermore, in addition to these aesthetic judgment criteria, we also added several that we identified as potentially relevant in the context of contemporary music and are also discussed in the aesthetics and philosophy of art literature: Interest (Items 15 and 16, Silvia, 2005; Emerson and Egermann, 2018), Entertainment (Item 17, Shusterman, 2003), and Intellectual Challenge (Item 18, Gaut, 2000). We also added assessments of the overall aesthetic and artistic value of the music (‘*I found the music to be aesthetically valuable’ and ‘I found the music to be artistically valuable’)* in order to validate the measurements made with aesthetic judgment criteria. The aesthetic judgment and emotion questionnaires were filled in retrospectively after each piece of music was presented.

**TABLE 2:** Aesthetic judgment criteria items and categories used in questionnaires.

**Item number**	**Item wording**	**Criteria category**	**Category origin**
1	I found the music original.	Originality/Novelty	Juslin, 2013
2	I found the music expressive.	Expressivity	Juslin, 2013
3	I found the music skilfully performed.	Skill	Juslin, 2013
4	I found the music skilfully composed.	Skill	Juslin, 2013
5	I found the music communicating a message.	Message	Juslin, 2013
6	I found the music meaningful.	Message	Juslin, 2013
7	How well did you understand this piece?	Message	Juslin, 2013
8	I found the music typical of its genre.	Typicality/Style	Juslin and Västfjäll, 2008
9	I found the music fit within my previous ideas about music and art.	Typicality/Style	Juslin and Västfjäll, 2008
10	I found the music emotionally moving.	Emotion	Juslin, 2013
11	I found the music beautiful.	Beauty/Sublime	Juslin, 2013
12	I found it ugly.*	Beauty/Sublime	Juslin, 2013
13	I found it sublime.*	Beauty/Sublime	Juslin, 2013
14	I found it distasteful.*	Taste	Juslin and Isaksson, 2014
15	I found the music interesting.	Interest	Silvia, 2005
16	Made me curious.*	Interest	Silvia, 2005
17	I found the music entertaining.	Entertainment	Shusterman, 2003
18	I found the music intellectually challenging.	Challenge	Gaut, 2000

Notes: *Items were taken from the AESTHEMOS only used in Concert 2, Schindler et al., 2017).

For Concert 2, we decided to choose a more complex emotion questionnaire that included more varied types of negative emotions. We therefore choose the 42-item AESTHEMOS (Schindler et al., 2017). Since Items 12, 13, 14, and 16 (Table 2) from this scale reflected aesthetic judgments rather than emotions, we used them in corresponding analyses of aesthetic judgments (see section “RESULTS”). Questionnaires (which also collected various socio-demographic background variables) were presented to participants in both concerts via an iPad Mini, using the online survey platform Qualtrics.

#### Activation of the Peripheral Nervous System

In both concerts, physiological arousal measurements were collected with Shimmer GSR + sensors that were attached to participants non-dominant arm wrists; the data was recorded into each individual device’s internal SD card (Sample rate for Concert 1: 128 Hz, Concert 2: 256 Hz). We attached an optical ear lobe sensor (photoplethysmography) to their non-dominant’s side ear recording blood volume pulse, and the two GSR electrodes were placed on the same side’s proximal phalanges of the index and middle finger.

#### Expressive Behavior

In Concert 1, we measured the electromyographic activity of two facial muscles typically associated with emotional valence (Zygomaticus Major representing smiling/positive emotion, and Corrugator supercilii representing frowning/negative emotion, Cacioppo et al., 1986). We employed Shimmer EMG sensors that were also placed on our participant’s upper arms (and recorded the data into each device’s internal SD card with 256 Hz sample rate). EMG electrodes were placed on the side of the face contralateral to the dominant hand (with positive and negative electrodes aligned with the respective muscles and the reference electrodes placed behind the nearest ear). In Concert 2, we recorded all participants’ faces with four Panasonic HD Cameras placed in front of the audience, however, due to some data loss we were not able to extract facial expression data from these recordings.

#### Audiovisual Recordings

Performances in both concerts were recorded with an HD video camera facing the performers for the entire duration of the experiment. The audio was captured with a stereo pair of microphones placed next to the camera, about two meters away from the stage.

#### Response Synchronization

In both concerts, all physiological data were recorded on Shimmer sensors (GSR and EMG) with a real word timestamp from a Windows PC laptop that was running Shimmer’s software ConsensysPro. We took an additional video recording of the laptop screen showing its real word time together with the surrounding audio in the concert hall. This recording allowed us to determine at what exact time the first note had sounded in each concert, which could then be used to synchronize physiological response recordings with the high-quality audio recording.

### Procedure

The procedure employed in both concerts was approved by the Ethics Committee of the Arts and Humanities Faculty, University of York. Prior to the experiments, participants were only informed that we would measure their responses to music performed in a live concert. We did not reveal the between-subjects design of the study and our focus on aesthetic judgment of contemporary music prior to the concerts. Participants arrived in the afternoon and registered for the experiment (including signing the consent form). We then split them randomly into two groups; participants in each group were then guided to two different seminar rooms where they were exposed to one of two 45-min long talks: one group attended a talk about the aesthetic value of the music that was presented in the subsequent concert (Concert 1 *n* = 21 Concert 2 *n* = 28), and the other group on an unrelated topic from social psychology as a control condition (Concert 1 *n* = 20; Concert 2 *n* = 25). Thereafter, participants went into the concert hall (Arthur Sykes Rymer Auditorium, University of York) where the electrodes were placed on their body, and where they were given an iPad mini. They then sat down in a predetermined seat and filled-in a short pre-concert questionnaire. Subsequently, there was a short announcement about the purpose of the experiment, and then the concert started. In Concert 1, we recorded 60 s of physiological baseline activity before each piece of music was performed, however, as we noted that this was quite strongly interfering with the flow of the concert, in Concert 2, we reduced this to one baseline recording of 60 s at the beginning of the concert. During physiological measurements (baseline and music performance) participants were instructed to put their hands with electrodes attached on their leg and to try to not move their body intensively (in order to avoid any movement artifacts in recordings). In both concerts, after the performance of each piece ended, participants filled in the emotion and aesthetic judgment questionnaires. After the concerts were finished, participants filled in a post-concert questionnaire and received a compensation (Concert 1: 10 GBP, Concert 2: 20 GBP)

### Data Analyses

#### Physiology

Preprocessing of all physiological signals recorded was done in Matlab (Mathworks, Version 9.05.0). First, we linearly interpolated all signals at the original sample rate. Then, we computed various response scores that summarized the time series data recorded per participant and piece. For skin conductance we computed first the *mean Skin Conductance Level (Mean SCL)*. We then low-pass filtered the signal at 0.3 Hz (in order to remove extraneous information using a linear phase filter based on the convolution of a 4^th^-order Butterworth filter impulse response also convolved with itself in time reverse in order to avoid phase shifting). We performed linear detrending on the corresponding recording, also in order to remove any negative trends over time with breakpoints every 60 seconds (that are caused by an accumulation of charge over time between the skin and sensor, see Salimpoor et al., 2009). From the resulting signal, we extracted the *number of non-specific Skin Conductance Responses per second* (NS-SCR/sec) and their *mean amplitude (Mean NS-SCR Amp).* We applied a low-pass filter to the blood volume pulse signal and then we extracted continuously interpolated HR in beats per minute (BPM) by inversing the inter-beat period (detected by identifying adjacent minima). This allowed us to calculate the *mean heart rate (mean HR)* and measures of time-based heart rate variability as the first order *standard deviation of the corresponding HR distribution* (*SD HR*, also referred to SD NN). For the EMG recordings captured in Concert 1, we applied a low-pass filter (120 Hz), a high-pass filter (25 Hz), then rectified and integrated each muscle signal separately.

We finally removed any linear trends over the course of the concert and individual differences in baseline physiological activity (baseline normalization) by subtracting from the filtered and extracted signals the mean baseline activity in the silent 40 s preceding each stimulus presentation (Concert 1) or the mean baseline recording before the concert (Concert 2).

We conducted subsequent inferential statistical analyses via hierarchical linear models in SPSS using the MIXED procedure. We used z-transformed predictor and outcome variables in order to estimate standardized beta-coefficients. We specified a residual covariance structure defining the participant ID as grouping variable, and music piece as repeated variable. We chose the best fitting covariance structure based on the smallest AIC values (comparing structures (1) diagonal, (2) compound symmetry, or (3) compound symmetry: heterogeneous). For physiological response scores, linear modeling analyses indicated that baseline-corrected data did not increase the number of significant predictors in linear models. We therefore decided to report non-baseline-corrected response scores. We suggest that the baseline recordings in both concerts were not long enough to be valid representations of physiological baseline activity.

## Results

### Factor Analyses of Aesthetic Judgment Criteria

We first identified if aesthetic judgment criteria could be grouped into several underlying factors that represent affective and cognitive judgment dimensions. Therefore, we subjected ratings on the aesthetic judgment criteria questionnaires from both concerts to exploratory factor analyses. We decided to employ varimax rotation, because we aimed for uncorrelated factor score variables for further analyses and used the Kaiser Criterion (min. Eigenvalue > 1) to decide how many factors were extracted.

In Concert 1, we removed the item ‘emotionally moving’ from the analyses as we thought it would be tautological to test if this item is related to other emotional response items. We subsequently checked difficulty and standard deviation of each item. Accordingly, the item “How well did you understand this piece?” was removed due to a low mean and standard deviation below 1. All remaining items were retained and entered into the factor analysis. The resulting factor matrix is shown in Table 3. The KMO measure of sampling adequacy was deemed high enough (KMO = 0.80), and the Bartlett’s test of Sphericity was significant (Chi-square (df = 66) = 828.5, *p* < 0.001). We labeled the first underlying factor *Analytical Value* (AnVal_C1), because it includes mostly items that are related to cognitive engagement with the music (e.g., generating interest, showing skill, being original). We identified a second factor that we labeled *Semantic Value* (SemVal_C1), as it represents judgments based on criteria that are related to the underlying meaning of the music (e.g., communicating a message, being meaningful, etc.). The third factor only had high loadings of two items that describe either how well the piece of music fits to previous ideas about music and its typicality. We labeled this factor *Typicality Value* (TypVal_C1).

**TABLE 3:** Factor loadings for exploratory factor analysis with varimax rotation of aesthetic judgment criteria from Concerts 1 and 2.

**Concert 1**			

***I found the music*…**	**Analytical Value (AnVal_C1)**	**Semantic Value (SemVal_C1)**	**Typicality Value (TypVal_C1)**

*interesting*	**0.72**	0.23	0.20
*entertaining*	**0.71**	0.29	0.13
*original*	**0.57**	−0.10	−0.16
*skilfully composed*	**0.52**	0.39	0.45
*intellectually challenging*	**0.50**	0.33	0.09
*skilfully performed*	**0.47**	0.22	0.26
*to communicate a message*	0.08	**0.79**	0.03
*meaningful*	0.24	**0.75**	0.30
*expressive*	**0.44**	**0.47**	**0.41**
*fits within my previous ideas about music and art*	0.21	0.15	**0.85**
*typical of its genre*	−0.04	0.08	**0.56**

**Concert 2**			

I found the music/it*…	**Analytical-Semantic Value (AnSemVal_C2)**	**Traditional Aesthetic Value (TrAesVal_C2)**	**Typicality Value (TypVal_C2)**

*original*	**0.73**	−0.05	0.10
*interesting*	**0.68**	0.39	0.31
*skilfully composed*	**0.61**	0.27	**0.44**
*expressive*	**0.60**	**0.41**	0.39
*meaningful*	**0.58**	0.39	**0.46**
*to communicate a message*	**0.57**	0.30	0.40
challenged me intellectually*	**0.56**	0.40	−0.15
*entertaining*	**0.54**	**0.48**	0.39
*skilfully performed*	0.40	0.13	0.29
beautiful*	0.25	**0.68**	0.24
ugly*	−0.07	−**0.63**	−0.48
sublime*	0.18	**0.54**	0.01
curious*	**0.48**	**0.49**	−0.08
*fits within my previous ideas about music and art*	0.10	0.15	**0.69**
*typical of its genre*	0.21	−0.06	**0.56**
distasteful *	−0.06	−0.48	−**0.51**

Notes: Extraction Method: Principal Axis Factoring, rotation method: Varimax with Kaiser Normalization, Factor loadings > 0.40 bold. *Items from the Aesthetic Emotions Scale (AESTHEMOS), Schindler et al., 2017).

The AESTHOMOS questionnaire, which was employed in Concert 2, featured several items that reflected aesthetic judgments rather than feeling states (‘beautiful,’ ‘sublime,’ ‘ugly,’ ‘distasteful,’ ‘challenged me intellectually,’ ‘Made me curious’). We therefore included those items in aesthetic judgment factor analyses in Concert 2 and not as emotional response measurements. Two AESTHEMOS items that were also included in our own self-developed aesthetic judgment criteria questionnaire (“Sparked my interest” and “Sensed a deeper meaning”) were not used in any analyses because they were already covered in our own list of the aesthetic judgment factors. We removed the items ‘emotionally moving’ (like in Concert 1), as well as the items “liked it”, “Was mentally engaged”, “Motivated me to act” and “Felt a sudden insight”, since according to our definition they represented neither aesthetic judgment criteria nor emotions. We subsequently checked difficulty and standard deviation of each item. None of the items had to be removed from further analyses and we therefore conducted the factor analyses with all remaining items. Table 3 presents factor loadings and shows that three aesthetic judgment factors (AJFs) were identified. The KMO measure of sampling adequacy was deemed high enough (KMO = 0.90), and the Bartlett’s test of Sphericity was significant (Chi-square (df = 120) = 3216.5, *p* < 0.001). Items representing the *Analytical* and *Semantic Value* of the music (see Concert 1) were grouped as one factor. This is why we labeled this factor *Analytical-Semantic Value* (AnSemVal_C2). Additionally, we identified a new factor representing judgment criteria that are usually associated with a traditional view of aesthetics (featuring items such as ‘beautiful’, ‘ugly’, ‘sublime’), and labeled it *Traditional Aesthetic Value* (TrAesVal_C2). We also identified the *Typicality Value* factor that we observed in Concert 1 in Concert 2 (TypVal_C2). We extracted factor scores from item ratings for both concerts using the regression method for use in subsequent analyses.

### Criterion Validity of Aesthetic Judgment Factors

We subsequently tested criterion validity of AJFs for measuring perceived value. AJFs scores were evaluated as predictors of audiences’ aesthetic and artistic value ratings (which were collected as manifest variables). Therefore, we estimated four hierarchical linear models, with value ratings from Concert 1 and 2, as outcome variables and AJFs as predictor variables (see Table 4). As can be seen in these results, in both concerts, all AJFs were significantly and positively associated with aesthetic as well as artistic value ratings. Furthermore, in Concert 1, *Semantic Value* (SemVal_C1) had the strongest influence on aesthetic value (compared to other factors), whereas artistic value was most strongly associated with *Analytical Value* (AnVal_C1). In Concert 2 however, *Analytical-Semantical Value* (AnSemVal_C2) was most strongly associated with aesthetic and artistic value (compared to the other two predictor variables).

**TABLE 4:** Hierarchical linear model of aesthetic judgment value factors as predictors of aesthetic and artistic value ratings.

	**Aesthetic Value Ratings**		**Artistic Value Ratings**	
**Predictor**	**β**	***SE*β**	**β**	***SE*β**
**Concert 1**				
Intercept	0.00	0.07	0.00	0.06
Analytical Value (AnVal_C1)	0.44	0.06***	0.54	0.06***
Semantic Value (SemVal_C1)	0.55	0.06***	0.42	0.06***
Typicality Value (TypVal_C1)	0.36	0.06***	0.37	0.06***
**Concert 2**				
Intercept	–0.01	0.04	0.00	0.04
Analytical-Semantic Value (AnSemVal_C2)	0.51	0.03***	0.64	0.03***
Traditional Aesthetic Value (TrAesVal_C2)	0.36	0.03***	0.27	0.03***
Typicality Value (TypVal_C2)	0.54	0.04***	0.48	0.04***

Notes: ***p < 0.001; Aesthetic and artistic value ratings were provided on one item each.

### Influence of Different Pieces of Music and Preconcert Talk on Aesthetic Judgment Factors

The six AJFs were subsequently tested to find how each of them were influenced by the preconcert talk and the different pieces of music presented in both concerts. Six hierarchical linear models were estimated, indicating that the musical piece variable significantly influenced all AJFs (see Table 5). However, neither the preconcert talk (Type of Talk) nor the interaction of piece of music with type of talk (Piece ^∗^ Type of Talk) had a significant effect on AJFs.

**TABLE 5:** Hierarchical linear models testing for effect of type of pre-concert talk and piece of music on aesthetic judgment value factors.

	**Concert 1**				**Concert 2**			
**Factor**	**df1**	**df2**	**F**	***P***	**df1**	**df2**	**F**	***p***

	**Analytical Value (AnVal_C1)**				**Analytical-Semantic Value (AnSemVal_C2)**			

Intercept	1.0	39.0	0.0	0.995	1.0	51.2	0.0	0.990
Piece	3.0	63.7	13.2	< 0.001	6.0	102.8	5.3	< 0.001
Type of Talk	1.0	39.0	0.1	0.787	1.0	51.2	0.1	0.823
Piece * Type of Talk	3.0	63.7	0.4	0.786	6.0	102.8	1.7	0.123

	**Semantic Value (SemVal_C1)**				**Classical Aesthetic Value (TrAesVal_C2)**			
Intercept	1.0	39.0	0.0	0.965	1.0	51.0	0.0	0.974
Piece	3.0	117.0	7.9	< 0.001	6.0	306.0	12.3	< 0.001
Type of Talk	1.0	39.0	3.2	0.082	1.0	51.0	0.3	0.565
Piece * Type of Talk	3.0	117.0	0.8	0.517	6.0	306.0	1.9	0.074

	**Typicality Value (TypVal_C1)**				**Typicality Value (TypVal_C2)**			

Intercept	1.0	39.0	0.0	0.991	1.0	51.0	0.0	0.959
Piece	3.0	117.0	21.5	< 0.001	6.0	306.0	29.9	< 0.001
Type of Talk	1.0	39.0	0.2	0.634	1.0	51.0	0.8	0.364
Piece * Type of Talk	3.0	117.0	0.2	0.916	6.0	306.0	1.4	0.221

**FIGURE 2:**
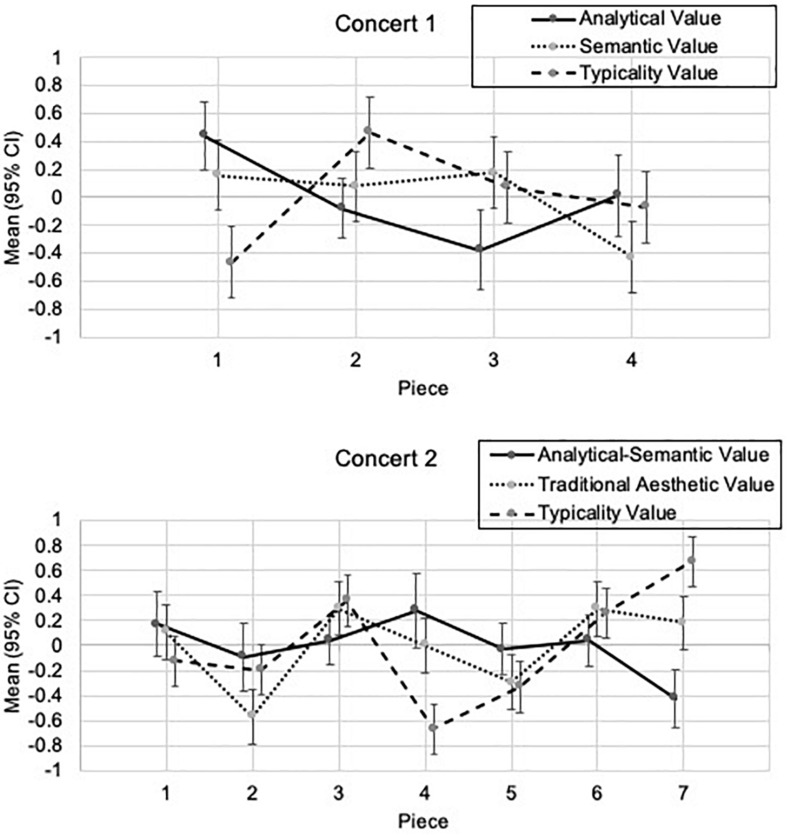
Predicted mean aesthetic judgment factor values separated by piece, concert, and value type.

As can be seen in Figure 2, in Concert 1, Piece Number 1 (Luck, Things) was considered to have rather low *Typicality Value* (TypVal_C1), however it received high scores for *Analytical* and *Semantic Values* (AnVal_C1, SemVal_C1). Piece Number 2 (Stockhausen, Klavierstück IX) was rated with high *Semantic Value* (SemVal_C1), and Piece Number 3 (Oliveros, Bye Bye Butterfly) and Piece Number 4 (free improvisation) received the lowest value ratings for *Semantic Value* (SemVal_C1).

Furthermore, in Concert 2, different pieces evoked different aesthetic value judgments. For instance, Piece Number 2 (Stockhausen, Klavierstücke VII) received the lowest *Traditional Aesthetic Value* ratings (TrAesVal_C2), and Piece Number 7 (Finnissy, Our Love Is Here To Stay) the highest. This last piece was also rated with the highest *Typicality Value* (TypVal_C2), whereas Guero from Lachenman was rated as the least typical (Piece 4).

### Relationships Between Pieces of Music, Aesthetic Judgment Factors and Subjective Feelings

After we established that AJFs were significantly influenced by the music that was presented to participants, we then evaluated if aesthetic judgments factors are in turn associated with ratings of subjective feelings. To increase the interpretability of results, we reduced the overall number of outcome variables representing subjective feelings. We therefore grouped the questionnaire items representing emotional qualities into various subgroups using exploratory factor analyses and used the Kaiser-Criterion (min. Eigenvalue > 1) to decide how many factors were extracted. For Concert 1, we identified three underlying factors: *Joyfulness, Sentimentality* and *Tension* (see Table 6).

**TABLE 6:** Factor loadings for exploratory factor analysis with varimax rotation of subjective feeling items (GEMS-25) from Concert 1.

***Please describe how the music you listened to made you feel.***	**Sentimentality**	**Joyfulness**	**Tension**
*tender*	**0.71**	0.15	0.02
*sad*	**0.69**	−0.15	0.34
*nostalgic*	**0.65**	0.20	−0.11
*mellowed (softened up)*	**0.63**	0.06	−0.31
*calm*	**0.58**	0.10	−0.35
*soothed*	**0.57**	0.19	−0.36
*tearful*	**0.56**	−0.08	0.21
*dreamy*	**0.53**	0.32	−0.20
*feeling of transcendence*	**0.50**	0.34	−0.07
*serene*	**0.50**	0.25	−0.29
*moved*	**0.50**	0.28	0.09
*affectionate*	**0.47**	**0.42**	0.03
*allured*	**0.46**	0.28	−0.05
*sentimental*	**0.45**	0.17	0.10
*energetic*	−0.02	**0.66**	0.38
*bouncy*	0.06	**0.62**	0.26
*triumphant*	0.17	**0.58**	0.03
*joyful*	0.27	**0.55**	−0.05
*strong*	0.16	**0.52**	0.18
*filled with wonder*	0.32	**0.50**	−0.19
*fascinated*	0.12	**0.50**	0.06
*animated*	0.10	**0.46**	0.41
*tense*	−0.08	0.17	**0.70**
*agitated*	−0.06	0.10	**0.60**
*overwhelmed*	0.02	0.28	0.40

*Extraction Method: Alpha Factoring, Rotation method: Varimax with Kaiser Normalization, Factor loadings > 0.40 bold, Items from Geneva Emotional Music Scale (GEMS-25), Zentner et al., 2008).*

For Concert 2, we identified five underlying factors: Joyful*ness, Sentimentality, Tension, Surprise*, and *Boredom* (see Table 7). Factor scores were calculated for datasets from both concerts using the regression method and subsequently used for further analyses.

**TABLE 7:** Factor loadings for exploratory factor analysis with varimax rotation of subjective feeling items (AESTHEMOS) from Concert 2.

***How intensely did you feel this emotion?***	**Joyfulness**	**Sentimentality**	**Tension**	**Surprise**	**Boredom**
*Made me happy*	**0.78**	0.21	−0.19	0.04	0.06
*Invigorated me*	**0.73**	0.15	0.17	−0.01	−0.08
*Energized me*	**0.73**	0.04	0.12	−0.05	−0.23
*Delight me*	**0.72**	0.39	−0.29	0.02	−0.09
*Fascinated me*	**0.71**	0.26	−0.09	0.24	−0.18
*Felt something wonderful*	**0.69**	0.46	−0.08	−0.01	−0.01
*Spurred me on*	**0.68**	0.14	0.28	−0.04	−0.03
*Amused me*	**0.66**	−0.01	−0.09	0.29	0.03
*Was impressed*	**0.65**	0.29	−0.10	0.12	−0.23
*Was enchanted*	**0.64**	0.45	−0.17	0.00	−0.10
*Felt awe*	**0.56**	0.30	0.12	0.02	−0.08
*Was funny to me*	**0.43**	−0.19	−0.04	0.38	0.19
*Made me feel sentimental*	0.24	**0.76**	−0.03	−0.13	−0.06
*Touched me*	0.45	**0.71**	−0.08	−0.09	−0.09
*Made me feel melancholic*	0.03	**0.70**	0.18	0.09	−0.09
*Felt deeply moved*	0.41	**0.69**	−0.02	−0.05	−0.15
*Made me feel nostalgic*	0.22	**0.68**	−0.03	−0.07	−0.07
*Made me sad*	−0.08	**0.68**	0.23	0.07	−0.18
*Calmed me*	0.25	**0.63**	−0.33	−0.04	0.24
*Relaxed me*	0.34	**0.57**	−0.27	−0.06	0.23
*Made me aggressive*	0.12	−0.12	**0.75**	−0.17	−0.06
*Was unsettling to me*	−0.06	−0.03	**0.64**	0.28	0.08
*Worried me*	−0.09	0.17	**0.63**	0.16	−0.15
*Made me angry*	−0.04	−0.12	**0.60**	−0.13	0.08
*Felt oppressive*	0.08	0.15	**0.59**	0.08	0.15
*Felt confused*	−0.07	−0.05	**0.53**	0.37	0.25
*Surprised me*	0.45	−0.05	0.19	**0.57**	−0.14
*Baffled me*	0.09	−0.07	0.46	**0.50**	0.22
*Bored me*	−0.40	−0.14	0.27	0.02	**0.49**
*Felt indifferent*	−0.20	−0.09	0.09	0.07	0.37

*Extraction Method: Alpha Factoring, Rotation method: Varimax with Kaiser Normalization, Factor loadings > 0.40 bold; Based on selection of items from The Aesthetic Emotions Scale (AESTHEMOS), Schindler et al., 2017).*

Subsequently, we tested if the resulting subjective feeling factors from Concert 1 and 2 could be predicted by the aesthetic judgment factor scores. We estimated one hierarchical linear model per dependent variable (see Table 8) and introduced the factor for piece of music as another independent variable (which was recoded to dummy variables with the last piece in the concert as reference category). This was done in order to control for the influence of other musical parameters that triggered other emotion induction mechanisms not related to aesthetic judgment. In both concerts, the pieces of music significantly influenced all outcome variables. Furthermore, individual differences in aesthetic judgments were also significantly related to subjective feeling factors.

**TABLE 8:** Hierarchical linear models of aesthetic judgment value factors and pieces of music as predictors of subjective feeling factors.

**Concert 1 (Subjective Feeling Factors based on GEMS)**										

	**Sentimentality**		**Joyfulness**		**Tension**					
**Predictor**	**β**	***SE*β**	**β**	***SE*β**	**β**	***SE*β**				

Intercept	–0.35	0.08***	0.31	0.13*	0.13	0.13				
[Piece = 1]^1^	0.22	0.11*	–0.31	0.13*	0.14	0.16				
[Piece = 2]^1^	0.52	0.12***	–0.44	0.13***	–0.23	0.16				
[Piece = 3]^1^	0.66	0.15***	–0.50	0.13***	–0.43	0.16**				
Analytical Value (AnVal_C1)	–0.14	0.06*	0.36	0.07***	–0.04	0.08				
Semantic Value (SemVal_C1)	0.34	0.06***	0.13	0.07^†^	0.06	0.08				
Typicality Value (TypVal_C1)	0.00	0.07	0.03	0.08	–0.28	0.09**				

**Concert 2 (Subjective Feeling Factors based on AESTHEMOS)**
	**Sentimentality**		**Joyfulness**		**Tension**		**Surprise**		**Boredom**	
**Predictor**	**β**	***SE*β**	**β**	***SE*β**	**β**	***SE*β**	**β**	***SE*β**	**β**	***SE*β**

Intercept	0.52	0.12***	–0.14	0.1	–0.20	0.09*	–0.29	0.09**	0.28	0.11*
[Piece = 1]^2^	–0.81	0.13***	0.37	0.12**	0.50	0.13***	0.32	0.13*	–0.52	0.14***
[Piece = 2]^2^	–0.46	0.13***	–0.06	0.11	0.36	0.13**	0.30	0.13*	–0.25	0.14†
[Piece = 3]^2^	0.13	0.15	–0.17	0.11	0.14	0.09	0.08	0.1	–0.38	0.13**
[Piece = 4]^2^	–0.94	0.14***	0.15	0.13	–0.15	0.13	0.92	0.14***	–0.05	0.15
[Piece = 5]^2^	–0.40	0.13**	–0.01	0.11	0.28	0.12*	0.34	0.14*	–0.24	0.14
[Piece = 6]^2^	–1.18	0.12***	0.73	0.11***	0.30	0.12*	0.04	0.11	–0.53	0.13***
Analytical-Semantic Value (AnSemVal_C2)	0.02	0.04	0.32	0.04***	0.16	0.04***	0.24	0.04***	–0.16	0.05**
Classical Aesthetic Value (TrAesVal_C2)	0.36	0.04***	0.57	0.04***	–0.38	0.05***	0.05	0.05	–0.18	0.05***
Typicality Value (TypVal_C2)	0.10	0.05*	–0.06	0.04	–0.27	0.05***	–0.18	0.05***	–0.18	0.05**

^1^ Dummy Coding with Piece = 4 as reference category; ^2^ Dummy Coding with Piece = 7 as reference category; ***p < 0.001, **p < 0.01, *p < 0.05, ^†^*p* < 0.10.

In Concert 1, *Analytical Value* (AnVal_C1) was negatively associated with sentimental feelings and positively with joyful feelings, indicating that it might have aroused and triggered positive feelings. *Semantic Value* (SemVal_C1) was positively associated with sentimental and joyful feelings, indicating that it might have created positive experiences independently from subjective arousal (for Joyfulness, we could observe however only a non-significant trend). High *Typicality Value* (TypVal_C1) in turn was associated with a reduction of negative experiences.

In Concert 2 these analyses indicated a rather similar picture: *Analytical-Semantic Value* (AnSemVal_C2) was mostly associated with feelings that contain arousal (positively with Joyfulness, Tension, Surprise, and negatively with Boredom). The new aesthetic judgment factor, *Traditional Aesthetic Value* (TrAesVal_C2), was positively associated with positive feelings (Sentimentality and Joyfulness) and negatively with negative feelings (Tension, Boredom), indicating that it might be related to the overall valence of the experience. Similar to Concert 1, high *Typicality Value* (TypVal_C2) was associated with a reduction in tense, surprised and bored experiences and an increase in Sentimentality.

### Relationships Between Pieces of Music, Aesthetic Judgment Factors, and Physiological Response Scores

In both concerts we estimated one hierarchical linear model for each physiological response score type (non-baseline-corrected). We employed a backward fitting strategy (West et al., 2007): first, by fitting full models with all predictors (piece dummy variables and aesthetic judgment factor scores). In a second iteration we removed all predictor variables from the models with *t*-values smaller than 1 (increasing the test power of resulting models with remaining predictor variables).

Generally, psychophysiological response scores reflecting arousal were significantly influenced by the different pieces of music (see Table 9). This indicates that the response scores recorded and calculated for this data systematically covary with musical characteristics representing different emotion induction mechanisms. Moreover, individual differences in aesthetic judgments were also significantly associated with physiological response scores.

**TABLE 9:** Hierarchical linear models of aesthetic judgment value factors and pieces of music as predictors of physiological response scores.

	**Mean SCL**		**NS-SCR/sec**		**Mean HR**		**SD HR (SDNN)**	
**Predictor**	**β**	***SE*β**	**β**	***SE*β**	**β**	***SE*β**	**β**	***SE*β**
**Concert 1**								
Intercept	0.01	0.16	–0.04	0.16	0.05	0.14	–0.10	0.13
[Piece = 1] ^1^	0.17	0.06**	–0.22	0.15	–0.06	0.06		
[Piece = 2] ^1^	–0.06	0.05	0.08	0.15	–0.05	0.05		
[Piece = 3] ^1^	–0.16	0.05**	0.29	0.15^†^	–0.08	0.04		
Analytical Value (AnVal_C1)	–0.06	0.03*			0.05	0.03^†^	–0.05	0.05
Semantic Value (SemVal_C1)	0.03	0.03	0.15	0.09^†^			–0.11	0.05*
Typicality Value (TypVal_C1)	0.03	0.04	–0.18	0.10^†^	–0.05	0.03		
**Concert 2**								
Intercept	0.07	0.15	–0.09	0.15	0.15	0.15	0.30	0.15*
[Piece = 1] ^2^	–0.17	0.05***	0.17	0.16	–0.15	0.05**	–0.69	0.10***
[Piece = 2] ^2^	–0.16	0.05**	0.14	0.17	–0.09	0.06	–0.40	0.11***
[Piece = 3] ^2^	–0.10	0.04*	–0.03	0.16	–0.06	0.05	–0.22	0.11^†^
[Piece = 4] ^2^	–0.01	0.05	0.24	0.16	–0.42	0.06***	–0.32	0.13*
[Piece = 5] ^2^	–0.09	0.05*	–0.02	0.16	–0.21	0.05***	–0.27	0.11*
[Piece = 6] ^2^	0.06	0.04	0.08	0.16	–0.11	0.05*	–0.21	0.10*
Analytical-Semantic Value (AnSemVal_C2)	0.00	0.02			0.03	0.02		
Classical Aesthetic Value (TrAesVal_C2)	0.01	0.02	0.15	0.07*				
Typicality Value (TypVal_C2)	–0.01	0.02					–0.07	0.05

^1^ Dummy Coding with Piece = 4 as reference category; ^2^ Dummy Coding with Piece = 7 as reference category; ***p < 0.001, **p < 0.01, *p < 0.05, ^†^*p* < 0.10.

In Concert 1, *Analytical Value* (AnVal_C1) was associated with a reduction in skin conductance response scores (Mean SCL) and an increase in heart rate (Mean HR, non-significant trend). Higher *Semantic Value* (SemVal_C1) judgments resulted in increased non-specific skin conductance responses per second (NS-SCR/sec, non-significant trend) and reduced heart rate variability response scores. *Typicality Value* (TypVal_C1) only showed a non-significant trend in being associated with a reduction of NS-SCR/sec. Facial expression recordings (representing zygomaticus major and corrugator muscle activations) from Concert 1 were not significantly associated with any of the predictor variables tested (not shown here).

In Concert 2, the combined *Analytical-Semantic Value* (AnSemVal_C2) and the Typicality (TypVal_C2) factors were not significantly associated with any response scores. However, the *Traditional Aesthetic Value* (TrAesVal_C2) was positively correlated with NS-SCR/sec.

## Discussion

The results presented in this study confirm several of the initially proposed hypotheses:

Ratings on the different aesthetic judgment criteria can be grouped into several underlying aesthetic judgment factors (AJFs): three factors represent cognitive value assessments of aesthetic and artistic qualities (*Analytical*, *Semantic*, and *Typicality* values), and one factor (that was only identified in Concert 2 as new judgment criteria were introduced into this experiment’s questionnaire) represents rather affective assessments of *Traditional Aesthetic* values including beauty, sublimity, and taste (H_1_)_._

All four AJFs were shown to be positively correlated with aesthetic and artistic value ratings of participants in both concerts (H_2_). In Concert 1, *Semantic Value* (SemVal_C1) was most strongly associated with aesthetic value, and *Analytical Value* (AnVal_C1) was associated with artistic value, indicating that they might represent two different value types (aesthetic and artistic). However, this pattern could not be observed in Concert 2, because as a result of the factor analyses, *Analytical* and *Semantic* values were grouped together as one factor (AnSemVal_C2). While, as expected, the *Traditional Aesthetic Value* (TrAesVal_C2) factor was strongly associated with aesthetic value ratings, there was no difference in how *Typicality Value* (TypVal_C2) was associated with aesthetic or artistic value ratings.

In both concerts, the different pieces of music were assessed with significantly different levels for all four AJFs. This finding is reflecting the influence of different musical attributes on aesthetic judgments, strengthening the validity AJF measurements taken in this study. However, the preconcert talks did not influence how participants rated AJFs (neither in general nor specifically by piece) contrary to what was expected (H_3_). The hypothesis that AJFs might mediate between the variable for type of talk and emotional response variables can be rejected, since the preconcert talks did not influence AJFs (H_4_).

Based on these results, it is possible to corroborate that AJFs are associated with activations in the subjective feelings and physiological arousal emotion response components (Scherer, 2005) (H_5_). These findings replicate those of Juslin and Västfjäll (2008), who showed that positive aesthetic judgments were positively associated with emotional intensity. However, as opposed to the study presented here, these authors did not test which type of aesthetic judgment is associated with which type of emotional quality and did not measure the physiological activation component of emotion. Furthermore, in the presented study, the associations between AJFs and emotional response components can be observed while controlling for the effect of musical parameters that might trigger other emotion induction mechanisms that are not related to aesthetic judgment (e.g., emotional contagion, musical expectation).

Relationships might be present because AJFs are causing and modulating the emotional responses which is what was hypothesized initially here (Juslin, 2013), or because aesthetic judgments and cognitive appraisals are the result of emotional responses (Allen et al., 2013; Schindler et al., 2017). Differentiating between different aesthetic judgment factor types that represent either affective or cognitive assessments of the music might help to understand if emotions are caused by aesthetic judgments or if aesthetic judgments are partially influenced by emotions. In Concert 2, the aesthetic judgment factor labeled *Traditional Aesthetic Value* was identified and correlated with affective assessments. This factor was generally associated with an increase in positive and a reduction in negative experiences. It was accompanied by a higher amount of skin conductance responses (representing phasic activity of the sympathetic nervous system, Dawson et al., 2007). It still remains, however, an open question if these value assessments related to *Traditional Aesthetic Value* are really the cause of emotional responses (Juslin, 2013), or if they rather represent the same aesthetic-affective response to the music (that may be caused by another unknown underlying variable on the inter-individual level representing a different emotion induction mechanism, e.g., evaluative conditioning, Juslin and Västfjäll, 2008).

On the other hand, *Analytical, Semantic*, and *Typicality Values* seem to represent cognitive assessments of the music performed in the concerts. All of these three AJFs also correlate with emotional response scores, a finding which indicates together with previous research a potential causal effect of AJFs on emotional responses. Aesthetic judgment could be similar cognitive (re)-appraisals which have been previously shown to induce and modulate emotions (Gross, 2002; Scherer, 2005). Appraising a piece of music as original (high *Analytical Value*) or meaningful (high *Semantic Value)* could be similar to the encounter of a goal-congruent event that triggers or modulates an appropriate emotional response cascade. Accordingly, higher assessments of *Analytical Value* (Concert 1), might lead to less sentimental and more joyful experiences, accompanied by a corresponding reduction in skin conductance level and increase in heart rate (which could indicate positive experiences, Koelsch and Jäncke, 2015). High *Semantic Value* in turn could lead to increases in sentimentality (presumably related to the semantic content associated with the music performed) accompanied with a reduction in heart rate variability, which has been previously shown to be negatively correlated with arousal (Koelsch and Jäncke, 2015). In Concert 2, *Analytical and Semantic Value* were combined into one factor and the results also show an increase in positive feelings (and decrease in negative feelings), however, no physiological correlates can be observed here. Finally, the aesthetic judgment factor Typicality Value led in both concerts to a reduction of negative feelings (Concerts 1 and 2) and heart rate variability (only Concert 2). This indicates that assessing art as typical might coincide with a reduction of negative responses in the listeners. Ratings of high *Typicality Value* might indicate the existence of mental representations in listeners allowing them to form expectations about how the music will evolve over time. Previous research and theories support the idea that musical expectations may play a causal role in inducing emotional responses to music (Huron, 2006; Egermann et al., 2013). Those who were not able to anticipate the musical structures presented to them (rating low typicality), had more negative responses due to expectation violations than those who were able to make predictions in the music (rating high typicality).

### Limitations and Outlook

In both concerts, the preconcert talk did not influence aesthetic value judgments by audience members. A possible explanation for this, is that a limited 45-min-long intervention might not long be enough and too limited in content in order to change audience judgments about unfamiliar contemporary music. Therefore, we were not able to verify in a between-subjects design if an increase in aesthetic judgment through a preconcert talk in turn changes emotional response measures. This study therefore does not present evidence for a causal influence of aesthetic judgment on emotional responses, but rather correlational. It might have also been that changes in aesthetic judgments were induced by emotional responses that were caused by other emotion induction mechanisms (e.g., violations of musical expectation that could lead to the experience of tension (Huron, 2006), which in turn is then judged to be of high semantic value). Future research should employ more elaborate ways to induce high aesthetic value judgments in audiences that could then, in turn, lead to changes in emotional responses to the music presented. We speculate that methods that could lead to increasing aesthetic value in audience members’ judgments might include long-term interventions that communicate the aesthetic value of contemporary music through a series of talks in a longitudinal study or more practical engagement through, for example, participation in rehearsals or being involved in the creation of the music (Gross and Pitts, 2016). Furthermore, employing research methods that include continuous assessments of aesthetic judgments and emotional responses through real-time rating interfaces (e.g., Egermann et al., 2013) would allow to test if changes in aesthetic judgments precede or follow changes in emotional responses.

We were able, nevertheless, to show in two concerts, which represent two-independently conducted experiments, that interindividual differences in aesthetic judgment (independent from the talk attended) were strongly related to emotional response scores. While in both concerts generally, an increase in aesthetic or artistic value was shown to be related to more positive, or less negative, emotions, there were some differences between concerts in which AJFs were associated differently with emotional response scores. There could be two possible explanations for these observations: First, we expanded the questionnaires employed in Concert 2 compared to those used in Concert 1 by using the AESTHEMOS questionnaire (Schindler et al., 2017). This was done to increase the range of different aesthetic judgment criteria and emotions captured. Second, we recruited a different type of audience for Concert 2 (compared to Concert 1 which also featured music students as participants). This was done because there was an indication in a preliminary analysis of data from Concert 1 (not shown here) that non-music students would respond stronger to the pre-concert talk (compared to music students). However, we believe that future research should explore interindividual differences in aesthetic judgments with larger and more diverse samples than those presented here.

While studying responses to contemporary music might be relevant for studying the link between aesthetic judgments and emotional responses, it still has to be demonstrated if the results reported in this study can be replicated with other, more common and less challenging, types of music.

## Conclusion

The findings reported in this study contribute to the understanding of how, and to what extent, a relationship exists between aesthetic judgment processes and emotional responses to music. Through factor analyses, we were able to illustrate that aesthetic judgments can be grouped into several underlying affective and cognitive dimensions. We found a trend for a distinction between aesthetic value, linked to affective criteria, and artistic value, associated with cognitive criteria. In two concerts, aesthetic judgments were strongly associated with subjective and physiological emotional response measures, indicating that they either were causing them, or were the result of them. Those results therefore exemplify the role of cognitive-affective interactions in processing of music stimuli. The effects of *Analytical*, *Semantic* and *Typicality* values shown in these results illustrate that assessing the aesthetic value of music differently, might change how one responds to it emotionally. Finally, finding ways in which, through accessing additional knowledge and information about the music, aesthetic value judgments could be shaped may help opening up unfamiliar music, that otherwise could be experienced as emotionally difficult, to new audiences.

## Data Availability Statement

The datasets generated for this study are available on request to the corresponding author.

## Ethics Statement

The studies involving human participants were reviewed and approved by Arts and Humanities Ethics Committee, University of York. The participants provided their written informed consent to participate in this study.

## Author Contributions

HE and FR designed experiments and conducted experiments. HE analyzed the data and created a first draft of the manuscript. Both authors contributed to the article and approved the submitted version.

## Conflict of Interest

The authors declare that the research was conducted in the absence of any commercial or financial relationships that could be construed as a potential conflict of interest.
